# The effects of mobile phone use on walking: a dual task study

**DOI:** 10.1186/s13104-019-4391-0

**Published:** 2019-06-21

**Authors:** Patrick Crowley, Pascal Madeleine, Nicolas Vuillerme

**Affiliations:** 1 Univ. Grenoble Alpes, AGEIS, Grenoble, France; 2 Sport Sciences, Department of Health Science and Technology, Aalborg University, Aalborg, Denmark; 3 The National Research Centre for the Work Environment, Copenhagen, Denmark; 4 Institut Universitaire de France, Paris, France

**Keywords:** Gait control, Locomotion, Distracted walking, Effect of technology, Accident risk, Multitasking

## Abstract

**Objectives:**

The aim of this study was to examine the effects of walking at different speeds while using a mobile phone on spatiotemporal stride parameters among young adults. Ten participants (7 male, 3 female; age = 24.7 ± 4.4 years, mean ± 1SD) completed 12 walking trials. Trials consisted of tasks performed at both normal and fast walking speeds—walking only, walking while texting, and walking while talking on a mobile phone. Gait velocity, stride length, cadence, and double support time were computed using data from accelerometers on either shoe.

**Results:**

The effects of distracted walking were not significantly larger when performed at a self-selected fast walking speed compared with a normal walking speed. However, walking while texting produced significant decreases in gait velocity, stride length, and cadence, with a significant increase in double support time at both walking speeds. Moreover texting increased the size of the relative variability of walking, observed through a significant increase in the coefficient of variation of cadence, stride length, and double support time. The observed changes may be suggestive of compromised balance when walking while texting regardless of walking speed. This may place the individual at a greater risk of, slips, trips and falls.

## Introduction

Walking while using a mobile phone affects both how we walk and how we interact with our environment [1–3]. The physical effects can typically manifest through decreased gait velocity, shortened stride length, [4–9] and increased duration of double support time [4, 7–9]. These physical changes can occur in parallel with change in attentional behaviours, such as; looking both ways when crossing the road and focusing attention on the oncoming traffic—in particular among young adults [10, 11]. However, the existence of large variation in the methodological designs and reported outcome measures among previous research has placed limitations on the interpretation of the synthesized results [2, 3]. The cited methodological limitations include; task characteristics (e.g. task complexity), task prioritization, adequate consideration for the multi-factorial nature and effects of the dual task (e.g. walking speed), as well as the appropriateness of the experimental environment [2, 3].

With this in mind, the aim of this study was to examine the effects of walking at different speeds while using a mobile phone on spatiotemporal stride parameters among young adults. We hypothesized that the effects of mobile phone use on walking will be significantly larger for fast speed walking when compared with self-selected normal speed walking, due to the combined effect of walking at a sub-optimal speed and the additional demands of a secondary task [1, 12]. Further, we hypothesized that the relative size of variability in gait would increase when walking while texting and talking on a mobile phone (i.e., under dual-task condition) compared with walking under single-task condition [7].

## Main text

### Participants

Ten healthy young adults participated (7 males, 3 females; age = 24.7 ± 4.4 years; height = 176 ± 5.4 cm; body mass = 71.9 ± 12.2 kg, mean ± 1SD). All reported normal/corrected-to-normal vision and no neurological or musculoskeletal disorders, or injuries that interfere with walking or texting. Participants reported regular mobile phone use (ranging from ‘*every 5* *min*’ to ‘*every hour*’) and the use of their current mobile phone for more than 1 month at the time of testing. Written informed consent was obtained from all participants prior to testing.

### Experimental protocol

Participants completed 12 walking trials. The following six conditions were repeated once by each participant: (1) normal walking speed (NWS) only, (2) NWS + texting, (3) NWS + talking on a phone, (4) fast walking speed (FWS) only, (5) FWS + texting, (6) FWS + talking on a phone. The order of the first six trials followed a randomized partial counterbalance design. Then, following a short break of approximately 5 min, this sequence was repeated. Testing was conducted along an 80 m indoor corridor. Participants were instructed to walk at the assigned pace (NWS or FWS) with no specified task prioritization. For example, the instruction before texting trials was: *“This time walk at a normal speed/at a fast speed, and attempt answer the questions received in the text message”.* Participants sent their responses in a single text at the end of each texting trial. Conversations during the talking trials covered the same topic as the texting trials. At baseline and following each trial, participants rated their perceived exertion (RPE) [13].

### Texting analysis

Texting performance was assessed at baseline. For this assessment, participants typed the pangram “The quick brown fox jumped over the lazy dog”. This was repeated three times and assessed using the average number of characters-per-second over the final two attempts. Texts received during the walking trials were also analysed using characters-per-second.

Questions covered the topics of sport, music, film, hobbies, food, and study program. (e.g. “*What is your favourite music genre and artist? Why is this genre your favourite? Why is this your favourite artist, give me three reasons? What is your favourite song by your favourite artist? How often do you listen to their music? What is your least favourite music genre?”*

### Gait analysis

Two Physilog 10D monitors (Gait Up SA, Lausanne, Switzerland), containing a tri-axial accelerometer, were attached over the laces of either shoe using an elastic strap. Raw accelerometer data was processed using Physilog Research ToolKit v1.1.1 (Gait Up SA, Lausanne, Switzerland), whereby signals were low pass filtered (17 Hz) and sampled at 200 Hz [14, 15].

The following four stride parameters and its corresponding coefficient of variation (CV) were computed:

- Walking speed (m/s), defined as the mean speed of forward velocity;
- Cadence (strides/min) was number of gait cycles in a minute;
- Stride length (m) was the distance between two successive footfalls, from the heel strike of one foot to the following heel strike of the same foot;
- Double support time (% of gait cycle) defined as the duration of the gait cycle during which both feet touch the ground [8].

As each walking trial was performed twice, a mean stride parameter value over both trials was calculated and used in the statistical analysis.

### Statistical analysis

Data distribution was assessed using Q–Q plots and Kolmogorov–Smirnov tests. Non-normal distributions underwent log transformation. The influence of walking speed (normal and fast), task (walking only; walking + texting; and walking + talking on a phone), and their interaction, on gait velocity, stride length, cadence, double support time, and RPE, was assessed using a within-subject repeated measures analysis of variance (2 × 3 RMANOVA). *α* was set at 0.05.

Post-hoc pairwise comparisons were made and adjusted using a Bonferroni correction, to reduce the potential bias introduced by multiple comparisons. A separate RMANOVA was used to assess the difference in characters typed per second, at baseline, while walking at a normal speed, and while walking at a fast speed. Analysis was completed using SPSS (IBM Statistics Data Editor V23). The observed power for all significant mean stride parameters results and CV values were above 80% and 65%, respectively.

### Results

Within-subject multivariate analysis showed a significant main effect of walking speed, task and speed × task interaction on mean stride parameters (Table 1). Subsequent univariate analyses showed a significant effect of walking speed on mean gait velocity, stride length, cadence and double support time (Table 1). Moreover, a significant effect of task was identified on each stride parameter. No significant interaction effect was evident at this point, indicating that, while both speed and task had an effect on stride parameters, the effect of task was not significantly larger at a fast walking speed. Pairwise comparisons indicated the varying effects of the three levels of task on the reported stride parameters. Walking while texting significantly decreased mean gait velocity, stride length, cadence and double support time (Table 2). Walking while talking on a phone significantly decreased mean stride length.

**Table 1:** Multivariate and univariate results of a RMANOVA on the reported spatiotemporal stride parameters

Multivariate		Mean values		Coefficient of variation values		
	Task	Speed	Speed × task	Task	Speed	Speed × task^a^
	Λ = 0.144 F_8,30_ = 6.1 *P *<* 0.001*	Λ = 0.087 F_4,6_ = 15.7 *P *=* 0.002*	Λ = 0.277 F_8,30_ = 3.4 *P *=* 0.007*	Λ = 0.011 F_8,30_ = 32.5 *P *<* 0.001*	Λ = 0.019 F_4,6_ = 76.7 *P *<* 0.001*	Λ = 0.012 F_8,30_ = 30.8 *P *<* 0.001*

Univariate	Task	Speed	Speed × task	Task	Speed	Speed × task^a^
Gait velocity	F_2,18_ = 23.9 *P *<* 0.001*	F_1,9_ = 71.5 *P *<* 0.001*	F_2,18_ = 0.8 P = 0.484	F_2,18_ = 2.5 P = 0.115	F_1,9_ = 122.8 *P *<* 0.001*	F_2,18_ = 65.2 *P *<* 0.001*
Cadence	F_1,18_ = 7.0 *P *=* 0.006*	F_1,9_ = 68.9 *P *<* 0.001*	F_2,18_ = 0.4 P = 0.668	F_1.1,9.9_ = 7.0 *P *=* 0.023*	F_1,9_ = 2.2 P = 0.170	F_2,18_ = 0.09 P = 0.914
Stride length	F_2,18_ = 38.8 *P *=* 0.000*	F_1,9_ = 62.6 *P *<* 0.001*	F_2,18_ = 1.0 P = 0.386	F_1.2,10.9_ = 19.9 *P *=* 0.001*	F_1,9_ = 68.3 *P *<* 0.001*	F_2,18_ = 29.9 *P *<* 0.001*
Double support time	F_2,18_ = 8.1 *P *=* 0.009*	F_1,9_ = 26.3 *P *<* 0.001*	F_1.2,10.7_ = 0.3 P = 0.638	F_2,18_ = 5.2 *P *=* 0.016*	F_1,9_ = 12.3 *P *=* 0.007*	F_2,18_ = 12.9 *P *<* 0.001*

Italic values represent significant findings

*Λ *=* Wilk’s Lambda*

^a^Significant values of this interaction effect are likely introduced by the log transformation of non-normally distributed variables

**Table 2:** Mean values ± 1SD of spatiotemporal stride parameters under each condition

Parameter	Single task		Dual-task			
	Walking only		Walking + texting		Walking + talking on a phone	
	NWS	FWS	NWS	FWS	NWS	FWS
Gait velocity (m/s)	1.4 ± 0.2	1.7 ± 0.2	1.3 ± 0.2^a^	1.5 ± 0.2^a^	1.4 ± 0.2	1.6 ± 0.2
Cadence (steps/min)	115 ± 6.3	126 ± 7.7	112 ± 8.5^a^	122 ± 9.1^a^	114 ± 7.8	124 ± 8.9
Stride length (m)	1.47 ± 0.12	1.61 ± 0.11	1.36 ± 0.13^a^	1.48 ± 0.12^a^	1.41 ± 0.09^b^	1.56 ± 0.10^b^
Double support time (% of gait cycle time)	20.9 ± 2.6	17.9 ± 2.4	22.9 ± 4.6^a^	20.6 ± 2.9^a^	22.0 ± 3.5	19.2 ± 2.5

Significant demarcation based on pairwise comparisons of the three levels of task

*NWS* self-selected walking speed, *FWS* self-selected fast walking speed

^a^Denotes ‘walking + texting’ condition differs significantly from the walking only condition at that speed (P < 0.05)

^b^Denotes ‘walking + talking on a phone’ condition differs significantly from the walking only condition at that speed (P < 0.05)

A further within-subject multivariate analysis of mean CV values also showed a significant effect of walking speed, task, and speed × task interaction (Table 1). Univariate analysis confirmed significant effects of speed on the CV of gait velocity, stride length, and double support time. Observed concurrently were significant effects of task on the CV of cadence, stride length, and double support time. With the exception of double support time while walking and talking on a phone, the mean CV increased with the addition of a dual task (Table 3). A significant interaction effect of speed × task on CV values is also reported (Table 1); however the subsequent assessment of the corresponding profile plots indicated that this interaction effect was introduced by the values of the log-transformed variables.

**Table 3:** Mean CV (%) values ± 1SD for each spatiotemporal stride parameter under each condition

Parameters	Single task		Dual-task			
	Walking only		Walking + texting		Walking + talking on a phone	
	NWS	FWS	NWS	FWS	NWS	FWS
Gait velocity	2.7 ± 0.8	3.1 ± 0.8	5.0 ± 1.8	5.2 ± 4.0	4.1 ± 1.5	3.8 ± 1.5
Cadence	1.7 ± 0.5	1.5 ± 0.4	2.6 ± 1.1^a^	2.4 ± 1.6^a^	2.2 ± 0.7^b^	2.0 ± 0.6^b^
Stride length	1.9 ± 0.6	2.1 ± 0.9	3.2 ± 1.0^a^	3.7 ± 3.8^a^	2.6 ± 0.8^b^	2.4 ± 0.6^b^
Double support time	8.0 ± 3.8	8.4 ± 4.4	9.7 ± 9.3^a^	8.9 ± 6.3^a^	7.6 ± 3.8	7.7 ± 3.9

Significance demarcation based on pairwise comparisons of the three levels of task

*NWS* self-selected walking speed, *FWS* self-selected fast walking speed

^a^Denotes ‘walking + texting’ condition differs significantly from the walking only condition (P < 0.05)

^b^Denotes ‘walking + talking on a phone’ condition differs significantly from the walking only condition (P < 0.05)

Mean RPE increased significantly from walking only trials (NWS = 8; FWS = 9) to walking while texting (NWS = 10; FWS = 11) and to walking while talking (NWS = 9; FWS = 10) trials respectively (P < 0.05). Mean texting performance at baseline was 2.7 ± 0.8 characters-per-second, which was significantly better than the mean texting performance during both NWS (1.2 ± 0.6) and FWS (1.2 ± 0.6) trials (P < 0.001).

### Discussion

We expected a larger effect of task at a fast walking speed when compared with a normal walking speed. Our findings did not support this hypothesis; instead we observed similar effect of task at both fast and normal walking speeds. Thereby, at both walking speeds, we observed a significant effect of walking while texting on each of the reported mean stride parameters. By contrast, only mean stride length changed significantly when walking while talking on a phone.

Actual texting performance during walking trials also declined compared with baseline, regardless of walking speed. However, while RPE scores increased on average for distracted walking trials and even more so at a fast walking speed, when compared with the single task condition, there was no statistically significant interaction effect on RPE (P = 0.052).

The bases for our primary hypothesis were the additional demand on resources placed by the combination of a sub-optimal walking speed and the cognitive demands of the distracting task. It appears that, in a group of young healthy adults at least, the challenge of walking while texting at fast walking speed produces changes in spatiotemporal gait parameters similar to those observed at normal walking speed despite a slight increase in the perceived exertion. In the present study, the increase in walking speed was approximately 17% so it is possible that larger increases in walking speed would have resulted in significant changes in the computed spatiotemporal gait parameters.

In agreement with our second hypothesis, the relative size of variability of gait was larger when walking while texting and talking on a phone were compared with walking only. The interpretation of variability in human walking most often falls under two categories: (1) arising as a result of the natural multiple degrees of freedom of human walking or (2) as increased instability in the system [16]. In the current study, the observed changes may be explained through the use of attentional models describing limited processing capacity during multiple task performance [17–19].

So why do the observed changes predominantly occur when walking while texting as opposed to when walking while talking on a phone? Texting requires the participant to focus on the screen while paying attention to the environment (e.g. direction of walking). This is not the case with walking while talking on a phone [20]. Here, although walking and talking are occurring simultaneously, the head position is typically upright, with the eyes free to take in the environmental information around. Following the multiple resources theory, visual channels can be broken down into two facets, focal and ambient [21]. Walking while texting, therefore, appears to require a large amount of processing both dimensions, whereas walking while talking would intuitively require less. When attentional resources are required to a large extent along one dimension the allocation of attention to other tasks is diminished [20].

We reported stride parameters deemed critical to stability [3, 22–26], therefore we interpret changes to these parameters as having a potential impact on gait stability. To use double support time as an example, as balance becomes compromised, there is a shift in the ratio between swing time and stance time in the favour of stance time to increase the time during which the feet are in contact with the ground [27]. As such, distracted walking may be linked to serious consequences such as slips, trips, and falls regardless of walking speed-even among a young demographic with no cognitive impairment [28, 29].

To conclude, the present study observed no significant difference in the effects of walking speed when comparing walking while texting, walking while talking on a phone and walking only. Still, our findings underline significant reductions in; gait velocity, cadence, and stride length concurrent with a significant increase in double support time, when walking while distracted.

## Limitations

The use of more advanced signal processing methods to assess gait may provide a more complete picture of the structural aspects of variability [30]. Future studies should apply advanced methods to better understand the influence of walking speed. Our results are constrained to the age range of our participants. Further, due to the small sample size; there is the potential for underpowered analysis; however the observed power of our results was above 65%.

## Data Availability

The datasets used and analysed during the current study are available from the corresponding author upon reasonable request to the corresponding author.

## Abbreviations

NWS: normal walking speed
FWS: fast walking speed
RPE: rating of perceived exertion
SD: standard deviation
CV: coefficient of variation
